# Coronal plane alignment changes do not affect outcome for total knee implant design with 3 degree varus joint line

**DOI:** 10.1186/s13018-025-05709-z

**Published:** 2025-04-04

**Authors:** Joshua Yeuk-Shun Tran, Esther Oi-Ching Chan, Cham-Kit Wong, Gloria Yan-Ting Lam, Tsz-Lung Choi, Rex Wang-Fung Mak, Jonathan Patrick Ng, Kevin Ki-Wai Ho, Patrick Shu-Hang Yung, Michael Tim-Yun Ong

**Affiliations:** 1 Department of Orthopaedics and Traumatology, The Chinese University of Hong Kong, Hong Kong SAR, China; 2 Department of Orthopaedics & Traumatology, Alice Ho Miu Ling Nethersole Hospital, Hong Kong SAR, China; 3 Department of Orthopaedics & Traumatology, Prince of Wales Hospital, Hong Kong SAR, China; 4 Department of Orthopaedics & Traumatology, CUHK Medical Centre, Hong Kong SAR, China

## Abstract

**Background:**

Total knee arthroplasty (TKA) is a widely performed surgical intervention for symptomatic knee osteoarthritis (OA). However, the influence of coronal plane alignment on clinical outcomes remains unclear. This study evaluates whether alterations in the coronal plane alignment of the knee (CPAK) classification, using the same implant system, affect postoperative outcomes in patients undergoing robotic-assisted TKA.

**Methods:**

We conducted a retrospective review of 202 patients who underwent primary robotic-assisted TKA between 2019 and 2023 using NAVIO or CORI systems and Journey II implants. Patients were classified based on changes in CPAK classification and functional outcomes assessed using the Knee Society Score (KSS) and Knee Society Function Score (KSFS) at multiple postoperative time points. Statistical analyses compared outcomes between groups with changed and unchanged CPAK.

**Results:**

A total of 202 patients were included. Preoperative demographic characteristics were comparable between groups. Functional outcome scores at 6 weeks, 3 months, 6 months, and 12 months postoperatively showed no significant differences between the changed CPAK group and the unchanged CPAK group (*p* > 0.05 for all comparisons). Subgroup analyses further revealed no statistically significant disparities in functional outcomes based on the type or degree of CPAK changes.

**Conclusion:**

Our findings suggest that changes in coronal plane alignment do not adversely affect functional outcomes following robotic-assisted TKA. This implies that alignment strategy may be less critical to patient satisfaction and functional recovery than previously assumed. Implant-induced alignment changes may contribute to comparable clinical outcomes and could be a key factor in patient recovery. Understanding the relationship between CPAK changes and clinical outcomes can inform best practices in knee arthroplasty, ultimately enhancing patient satisfaction and quality of life post-surgery.

## Introduction

Total knee arthroplasty (TKA) is one of the most frequently performed operations and has been shown to significantly improve functional outcomes and quality of life for patients with symptomatic knee osteoarthritis (OA). The prevalence of knee OA, the primary indication for TKA, increases markedly with age and rises almost linearly after 40 years of age [1]. A nationwide population-based study in China reported an 8.1% incidence rate of symptomatic knee OA in 2015, with prevalence increasing with age [2]. In the United States, over 32.5 million people are affected by OA, particularly those over 45 years old [3]. Globally, the pooled global prevalence of knee OA was 16% among individuals aged 15 and older and 22.9% among those aged 40 and over [4]. While TKA effectively alleviates pain and restores mobility in OA patients, optimizing surgical approach and knee alignment techniques remains a primary research focus. Accurate knee alignment is crucial, as it is associated with disease progression, functional decline and postoperative functional outcome [5]. Recent advancements in imageless robotic-assisted TKA systems, including the CORI and NAVIO Surgical Systems by Smith & Nephew, have improved alignment accuracy through real-time virtual 3D planning tailored to patients’ unique knee anatomy [6].

The coronal plane alignment of the knee (CPAK) classification system has emerged as a structured framework to better categorize patients’ knee alignment pre- and post-operatively [7]. CPAK classifies patients into one of nine groups based on measurements of the medial proximal tibial angle (MPTA) and lateral distal femoral angle (LDFA). Joint line obliquity (JLO) and arithmetic hip-knee-ankle angle (aHKA) are derived from these measurements, enabling assessment of coronal plane deformity type and severity. This system also supports preoperative planning for appropriate surgical strategy and implant selection, guides intraoperative alignment restoration and evaluates postoperative outcome.

However, the clinical impact of altering a patient’s native joint line and CPAK classification remains controversial. Agarwal et al. found that modifying the native joint line does not significantly affect postoperative satisfaction [8]. Similarly, Al-Abbasi et al. reported that no demonstrable difference in patient-reported outcome measures (PROMs) and survivorship related to the change in phenotype [9]. On the contrary, Konishi et al. concluded that changes in varus/valgus alignment negatively predicted outcomes on both the Knee Injury and Osteoarthritis Outcome Score (KOOS-12) and the Forgotten Joint Score (FJS-12) [10]. These mixed findings underscore the complexity of knee biomechanics and the lack of consensus on alignment standards in TKA.

Implant design philosophies vary across manufacturers, with recent designs focusing on replicating natural knee biomechanics. Multiple factors including range of motion, modularity, insert fixation and geometrical congruence between articulating surfaces are critical considerations. While early knee implant designs focused on achieving stability and durability, the recent evolution of knee designs aimed to preserve physiological knee motion and incorporate more anatomical features. The Journey II System (Smith & Nephew), for instance, aims to restore the midline anterior–posterior position and 3° varus joint line found in healthy knees [12]. This system potentially improves ligament tension and reduces paradoxical motion to better accommodate the biomechanical variations in individuals [12].

Historically, neutral mechanically aligned (MA) TKA has been the standard approach in TKA, involving the placement of implants perpendicular to the mechanical axis of both the tibia and femur [13]. While MA has been shown to promote implant survivorship and favourable clinical outcomes, it may not accurately replicate the native knee alignment and joint line orientation unique to each individual, which often deviates from the neutral mechanical axis. Kinematic alignment (KA) has emerged as a potential alternative, aiming to restore the patient’s natural and pre-arthritic alignment, thereby potentially improving gait and overall knee function [14]. Additionally, implant design and the alignment changes induced by specific implants are critical considerations in TKA. This retrospective study evaluates whether changes in CPAK classification, particularly those influenced by implant-induced alignment changes, impact clinical outcomes.

## Methods

### Study design

This retrospective analysis utilized prospectively collected data from patients who underwent primary robotic-assisted TKA at a tertiary center by the same team of experienced specialist orthopaedic surgeons from the arthroplasty division between 2019 and 2023. Data were obtained from the institutional joint registry and ethical approval was granted by the local research ethics committee.

### Patient selection

Patients who underwent primary TKA using the NAVIO or CORI robotic systems (Smith and Nephew, USA) were included in the study. The implants used were the Bi-cruciate Stabilized, Cruciate Retaining, and Bi-cruciate Retaining Journey II System (Smith and Nephew, USA). Exclusion criteria included 1) prior surgery on the same knee, including previous knee arthroplasty or osteotomy, 2) underlying disease or complicating conditions, such as previous periarticular fracture, severe fixed flexion contracture > 20°, multi-ligament instability, bone stock deficiency requiring augmentation and stems, neuromuscular disorder, acute and chronic infection, 3) absence of preoperative or postoperative long leg radiographs or patients lost to follow-up.

### Technique

All TKA surgeries were performed using either mechanical or kinematic alignment, based on the surgeon's preference. Identical wound closure techniques and postoperative recovery protocol, including perioperative analgesic and antiemesis measures, were implemented as part of the adult joint reconstruction enhanced recovery after surgery protocol. A standardized physiotherapy rehabilitation protocol for adult joint reconstruction was followed and patients were discharged once their mobility allowed outpatient care.

### Radiographic measurements

**Fig. 1:**
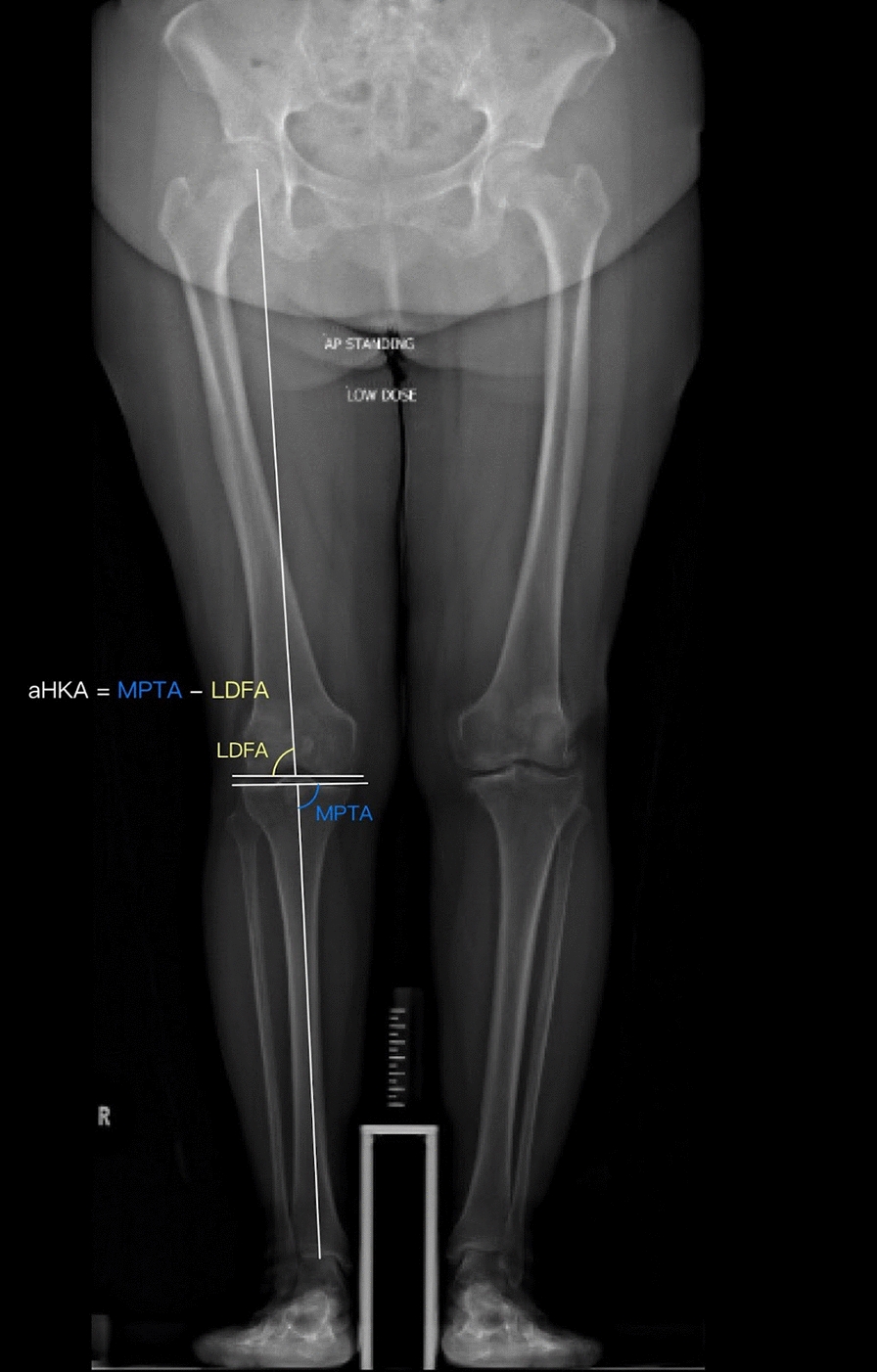
Radiographic parameters

Clinical data collected included the patients' demographic data, operation records, preoperative and postoperative functional scores and radiographs. Preoperative and postoperative radiographs were reviewed by two independent reviewers. Radiographic measurements, including the arithmetic hip-knee-ankle angle (aHKA), joint line obliquity (JLO) lateral distal femoral angle (LDFA) and medial proximal tibial angle (MPTA), were taken (Fig. 1). LDFA was measured as the angle between the femoral mechanical axis and the line tangent to the articular surface of the distal femur. MPTA was measured as the medial angle between the tibial articular marginal line and the mechanical axis from the ankle center to the center of the tibial spines [15]. Preoperative aHKA was calculated using the formula: aHKA = MPTA − LDFA. JLO was defined as the sum of MPTA and LDFA. The correlation of CPAK with clinical outcomes was then assessed. Interobserver reliability was evaluated by comparing the radiological measurements on the same set of radiographs between two independent reviewers. CPAK changes were then categorized into four groups based on changes in joint line, coronal plane alignment, combined joint line and alignment and CPAK transition specific to the Journey II implant design.

### Power analysis

A G*Power analysis was conducted based on the sample size. Independent T-Test was selected with a post hoc power analysis to compute achieved power. Given a two tailed input, an effect size of 0.5, and an α of 0.05, the calculated power is 0.8626.

### Statistical analysis

Patient demographic characteristics including age, sex, BMI, preoperative range of motion, and the side of operation, were compared between the patients with and without CPAK changes using Student's T-test or Chi-square test. Knee Society Knee Score (KSS) and Knee Society Function Score (KSFS) at pre-operation, 6 weeks, 3 months, 6 months, and 12 months post-operation were compared between the two groups using Student's T-test. Subgroup analysis was conducted on classifications of CPAK changes and KSS and KSFS were compared between patients changes using Student's T-test. For all analyses, *p* values of less than 0.05 were considered statistically significant. All statistical analyses were performed by using IBM SPSS version 28 (Armonk, NY: IBM Corp).

## Results

A total of 202 patients were recruited into the study. 152 patients in the changed coronal plane alignment (CPAK) group and 50 patients in the unchanged CPAK group were included. The pre-operative demographic characteristics, including age, BMI and pre-operative functional scores (KSFS and KSS), were comparable between the two groups, with no statistically significant differences (Table 1). The mean age was 70.9 ± 7.5 years for the changed CPAK group and 70.9 ± 9.1 years for the same CPAK group. There was 69.1% of females in the changed CPAK group and 80.0% in the same CPAK group. 46.1% of the cases were left knees in the changed CPAK group while 46.0% of the cases were left knees in the same CPAK group.

**Table 1:** Patient demographics

	Changed CPAK (n = 152)	Same CPAK (n = 50)	*p* value (95% CI)
Mean ± SD			
Age	70.9 ± 7.5	70.9 ± 9.1	0.996 (−2.6, 2.5)
BMI	27.6 ± 4.2	27.4 ± 4.0	0.774 (−1.1, 1.5)
KSFS Pre-Op	56.1 ± 19.9	54.8 ± 19.8	0.704 (−5.6, 8.2)
KSS Pre-Op	57.9 ± 19.3	54.7 ± 21.2	0.356 (−3.6, 10.0)
n (%)			
Sex (female)	105 (69.1%)	40 (80.0%)	0.137
Side (left)	79 (46.1%)	23 (46.0%)	0.464

Functional outcomes at 6 weeks, 3 months, 6 months, and 12 months post-operatively were assessed using KSFS and KSS (Table 2). At 6 weeks, KSS scores were nearly identical between the changed CPAK group (85.8 ± 12.9) and the same CPAK group (85.5 ± 10.5, *p* = 0.899). Similarly, at 3 months, the KSS scores were comparable, with 88.3 ± 10.5 in the changed CPAK group and 88.6 ± 12.1 in the same CPAK group (*p* = 0.899). By 12 months, KSSS scores also showed minimal difference between the two groups, with 94.3 ± 6.4 in the changed CPAK group and 91.7 ± 9.5 in the same CPAK group (*p* = 0.141). These findings indicate that functional scores were consistent across groups regardless of changes in coronal plane alignment.

**Table 2:** Post-operative functional scores—Student’s T-Test

	Changed CPAK (n = 152)	Same CPAK (n = 50)	*p* value (95% CI)
Mean ± SD			
KSFS 6 weeks	52.5 ± 24.6	44.2 ± 25.9	0.100 (−1.6, 18.2)
KSS 6 weeks	85.8 ± 12.9	85.5 ± 10.5	0.899 (−4.4, 5.0)
KSFS 3 months	64.3 ± 23.5	59.5 ± 23.9	0.339 (−5.1, 14.8)
KSS 3 months	88.3 ± 10.5	88.6 ± 12.1	0.899 (−4.8, 4.2)
KSFS 6 months	72.0 ± 20.0	68.2 ± 19.3	0.327 (−3.8, 11.4)
KSS 6 months	93.1 ± 7.9	90.0 ± 12.0	0.167 (−0.4, 6.5)
KSFS 12 months	76.8 ± 20.2	76.6 ± 19.0	0.945 (−7.3, 7.9)
KSS 12 months	94.3 ± 6.4	91.7 ± 9.5	0.141 (−0.2, 5.3)

Subgroup analyses were conducted to explore the impact of CPAK changes on functional outcomes to different extents. Patients were categorized into four subgroups based on the nature of changes observed in joint line, alignment, both combined and specific to Journey II implant design. The first subgroup compared patients with unchanged joint line to those with changed joint line (Table 3). The second subgroup compared patients with unchanged coronal plane alignment with those with changed coronal plane alignment (Table 4). The third subgroup evaluated the combined effect of changes in both coronal plane alignment and joint line, contrasting patients with unchanged CPAK phenotype to those with changed in both alignment and joint line (Table 5). The fourth subgroup targeted specifically on the Journey III implant design, comparing patients with CPAK phenotype changed from types 4–9 to types 1–3 with those who remained consistently in types 1–3 (Table 6). Overall, subgroup analysis shows no statistically significant difference in functional scores. Additional subgroup analysis was done (Appendix).

**Table 3:** Subgroup analysis of patients with changed joint line to those with unchanged joint line

	Changed Joint line (n = 16)	Same joint line (n = 44)	*p* value (95% CI)
Mean ± SD			
KSFS 0 weeks	52.8 ± 26.6	54.8 ± 19.8	0.753 (− 10.7, 14.7)
KSS 0 weeks	61.8 ± 14.1	54.7 ± 21.2	0.223 (− 18.5, 4.4)
KSFS 6 weeks	53.7 ± 24.9	44.2 ± 25.9	0.230 (− 25.3, 6.2)
KSS 6 weeks	87.2 ± 10.5	85.5 ± 10.5	0.586 (− 8.1, 4.6)
KSFS 3 months	70.0 ± 25.8	59.5 ± 23.9	0.204 (− 26.9, 5.9)
KSS 3 months	91.5 ± 8.8	88.6 ± 12.1	0.431 (− 10.5, 4.5)
KSFS 6 months	78.2 ± 15.1	68.2 ± 19.3	0.091 (− 21.6, 1.6)
KSS 6 months	88.4 ± 14.8	90.0 ± 12.0	0.684 (− 6.6, 9.9)
KSFS 12 months	81.1 ± 23.9	76.6 ± 19.0	0.489 (− 17.5, 8.5)
KSS 12 months	91.5 ± 10.2	91.7 ± 9.5	0.952 (− 6.0, 6.4)

**Table 4:** Subgroup analysis of patients with changed coronal plane to those with unchanged coronal plane

	Changed coronal plane (n = 66)	Same coronal plane (n = 44)	*p* value (95% CI)
Mean ± SD			
KSFS 0 weeks	60.3 ± 17.4	54.8 ± 19.8	0.128 (− 12.6, 1.6)
KSS 0 weeks	57.5 ± 20.2	54.7 ± 21.2	0.482 (− 10.7, 5.1)
KSFS 6 weeks	51.3 ± 25.8	44.2 ± 25.9	0.223 (− 18.5, 4.4)
KSS 6 weeks	86.1 ± 12.8	85.5 ± 10.5	0.791 (− 5.7, 4.4)
KSFS 3 months	65.2 ± 21.2	59.5 ± 23.9	0.283 (− 16.1, 4.8)
KSS 3 months	89.1 ± 8.8	88.6 ± 12.1	0.838 (− 5.2, 4.2)
KSFS 6 months	73.3 ± 20.3	68.2 ± 19.3	0.236 (− 13.4, 3.3)
KSS 6 months	93.8 ± 6.1	90.0 ± 12.0	0.097 (− 8.2, 0.7)
KSFS 12 months	78.6 ± 18.9	76.6 ± 19.0	0.607 (− 9.9, 5.8)
KSS 12 months	95.0 ± 5.6	91.7 ± 9.5	0.063 (− 6.9, 0.2)

**Table 5:** Subgroup analysis of patients with changed coronal plane alignment and joint line to those with unchanged CPAK phenotype

	Changed Joint line and coronal plane alignment (n = 36)	Same CPAK (n = 44)	*p* value (95% CI)
Mean ± SD			
KSFS 0 weeks	50.1 ± 21.3	54.8 ± 19.8	0.306 (− 4.4, 13.9)
KSS 0 weeks	56.1 ± 20.8	54.7 ± 21.2	0.760 (− 10.7, 7.8)
KSFS 6 weeks	55.1 ± 19.5	44.2 ± 25.9	0.067 (− 22.6, 0.8)
KSS 6 weeks	85.1 ± 13.5	85.5 ± 10.5	0.895 (− 5.5, 6.3)
KSFS 3 months	58.4 ± 25.5	59.5 ± 23.9	0.870 (− 12.3, 14.5)
KSS 3 months	84.5 ± 13.4	88.6 ± 12.1	0.242 (− 2.8, 11.0)
KSFS 6 months	69.4 ± 21.6	68.2 ± 19.3	0.817 (− 11.1, 8.8)
KSS 6 months	93.5 ± 6.9	90.0 ± 12.0	0.147 (− 8.3, 1.3)
KSFS 12 months	72.0 ± 21.7	76.6 ± 19.0	0.354 (− 5.2, 14.5)
KSS 12 months	93.3 ± 5.9	91.7 ± 9.5	0.409 (− 5.4, 2.2)

**Table 6:** Subgroup analysis of patients with CPAK phenotype changed from types 4–9 to types 1–3 with those who remained consistently in types 1–3

	Changed CPAK phenotype changed from types 4–9 to types 1–3 (n = 10)	Same CPAK in types 1–3 (n = 103)	*p* value (95% CI)
Mean ± SD			
KSFS 0 weeks	46.0 ± 24.1	58.5 ± 18.5	0.050 (0.02, 25.0)
KSS 0 weeks	59.8 ± 16.3	56.0 ± 20.5	0.550 (− 16.5, 8.8)
KSFS 6 weeks	50.5 ± 22.4	49.6 ± 26.1	0.913 (− 18.1, 16.2)
KSS 6 weeks	86.6 ± 10.4	85.7 ± 12.1	0.816 (− 8.9, 7.0)
KSFS 3 months	59.4 ± 15.9	63.6 ± 22.7	0.615 (− 12.3, 20.7)
KSS 3 months	91.6 ± 9.1	88.9 ± 10.2	0.499 (− 10.7, 5.2)
KSFS 6 months	72.7 ± 13.3	72.5 ± 18.7	0.969 (− 11.7, 11.3)
KSS 6 months	90.2 ± 16.0	92.3 ± 8.8	0.495 (− 4.0, 8.3)
KSFS 12 months	66.4 ± 19.1	78.9 ± 17.0	0.024 (1.7, 23.5)
KSS 12 months	92.8 ± 9.4	93.9 ± 7.23	0.636 (− 3.6, 5.8)

## Discussion

Our findings indicate that most patients have postoperative modification of their constitutional phenotype, which aligns with previous studies [17]. The discussion of CPAK classification system aims to refine the optimal alignment strategy for TKA [7]. This system provides a standardized framework for evaluating knee phenotypes, contributing to the ongoing debate on how alignment approaches impact clinical outcomes. This study is the first to investigate the impact of coronal plane alignment and joint line changes on clinical outcomes in patients undergoing imageless robotic-assisted TKA using the same implant system, while also examining implant-specific changes across subtypes.

The two primary alignment methods are mechanical alignment (MA) and kinematic alignment (KA). MA aims to align the knee to the mechanical axis, defined as the line connecting the centers of the femoral head and tibiotalar joint [18]. This method creates neutral coronal resections, adjusts apex distal JLO to neutral and externally rotates the femoral component, often resulting in alterations to the preoperative knee phenotype [19, 20]. MA is widely adopted due to its high reproducibility and ability to achieve balanced load distribution between the medial and lateral compartments, hence minimizing wear and potential component loosening [21]. Despite its excellent long-term implant survivorship [22], MA has been associated with suboptimal patient-reported outcomes (PROMS) including dissatisfaction and residual symptoms [23]. In contrast, KA aims to restore the patient’s native knee anatomy by replicating the preoperative CPAK phenotype and minimizing soft tissue releases. This approach relies on bone cuts to maintain ligamentous stability and knee kinematics [24]. However, the notion that a single alignment method suits all cases in TKA is often being challenged.

The role of CPAK classification in robotic-assisted TKA outcomes remains controversial. Research from Kyushu University identified alterations in varus/valgus alignment from preoperative to postoperative as a negative predictive factor for both KOOS-12 and FJS-12 scores [10]. Pangaud et al. reported that restoring CPAK phenotype improves PROMs results, including KOOS-12, Simple Knee Value and FJS-12 at 2 years of follow-up [17]. On the contrary, Sappey Marinier et al. found no significant difference in postoperative pain between patients with restored apex distal JLO and those non-restored [20]. Additionally, clinical and radiological results were similar between the KA and MA group [20]. Similarly, Sarang Agarwal et al. concluded that altering the patient’s native joint line and CPAK classification does not significantly impact surgical outcomes in terms of patient satisfaction. These mixed findings highlight the ongoing debate surrounding CPAK alterations.

The current study found similar clinical outcomes between changed and unchanged CPAK groups, potentially explained by the role of implant designs in eliminating alignment-related disparities in clinical outcomes. Subgroup analysis on changes across the joint line and coronal plane alignment showed comparable clinical outcomes, suggesting that varying degrees of alteration may not substantially affect postoperative functional outcomes. Similarly, changes in the joint line specific to the Journey II implant system showed no significant differences in functional outcomes, except for one isolated finding without a clear trend. This may be attributed to the implant’s design, which accommodates anatomical variations and maintains functional stability regardless of preoperative alignment. Implant designs enhance the precision of implantation of components through advanced instrumentation and technological assistance. The Journey II System (Smith & Nephew) used in this study may have contributed to functional optimization and comparable outcomes between KA and MA irrespective of alignment strategy. Journey II system was designed to restore the native 3° varus joint-line when the transverse axis of the artificial knee joint is perpendicular to the mechanical axis of lower limb, providing more normal ligament strain and patello-femoral tracking. A previous study found that 80.2% of knees have a distal femoral flexion angle with a mean of 3° ± 2° [24], suggesting that positioning the component in 3° of flexion from the mechanical axis would attain a satisfactory position [24]. Implant design and positioning play a critical role in determining patient-reported outcomes measure as misalignment is a known risk factor for poor outcomes [25]. This is further supported by the lack of difference between mobile bearing compared to fixed bearing implants in unicompartmental arthroplasty [26]. These findings underscore the importance of implant design and positioning in influencing clinical outcomes, independent of alignment strategy.

The study demonstrates that implant-induced changes in CPAK classification do not adversely impact clinical outcomes. To optimize the alignment strategy for patients, personalized implant design and deployment, along with assessments of constitutional bony anatomy and soft-tissue laxity, should be incorporated into intraoperative planning [27]. These considerations align with the emerging concept of functional alignment, which emphasizes tailoring implant sizing and positioning to balance soft-tissue laxity and restore constitutional bony alignment [28, 29].

Several limitations should be acknowledged. First, the retrospective nature of the analysis introduces potential biases, and the findings are limited to a single-centre cohort. Second, additional operative factors, including patella resurfacing [30], and long-term outcomes, including implant survivorship, should be evaluated to provide a comprehensive understanding. Third, the sample size of 202 patients is relatively small, limiting the generalizability of the findings. Future studies should include larger cohorts to reduce biases and enhance the external validity of the results.

## Conclusion

In summary, respecting the implant-induced change in CPAK may be more significant to clinical outcomes than solely preserving the pre-operative CPAK.

## Data Availability

No datasets were generated or analysed during the current study.

## Appendix

See Tables 7, 8, 9, and 10.

**Table 7:** Subgroup analysis of changed CPAK within same coronal plane

	Changed CPAK (n = 22)	Same CPAK (n = 50)	*p* value
Mean ± SD			
KSFS 6 weeks	53.8 ± 25.9	44.2 ± 25.9	0.230
KSS 6 weeks	87.2 ± 10.5	85.5 ± 10.5	0.586
KSFS 3 months	70.0 ± 25.8	59.5 ± 23.9	0.204
KSS 3 months	91.5 ± 8.8	88.6 ± 12.1	0.431
KSFS 6 months	78.2 ± 15.1	68.2 ± 19.3	0.091
KSS 6 months	88.4 ± 14.8	90.0 ± 12.0	0.684
KSFS 12 months	81.1 ± 23.9	76.6 ± 19.0	0.489
KSS 12 months	91.5 ± 10.2	91.7 ± 9.5	0.952

**Table 8:** Subgroup analysis of changed CPAK within different coronal plane

	Changed CPAK (n = 130)	Same CPAK (n = 50)	*p* value
Mean ± SD			
KSFS 6 weeks	52.3 ± 24.6	44.2 ± 25.9	0.118
KSS 6 weeks	85.5 ± 13.3	85.5 ± 10.5	0.978
KSFS 3 months	63.3 ± 23.9	59.5 ± 23.9	0.459
KSS 3 months	87.7 ± 10.7	88.6 ± 12.1	0.716
KSFS 6 months	71.2 ± 20.5	68.2 ± 19.3	0.458
KSS 6 months	93.8 ± 6.2	90.0 ± 12.0	0.093
KSFS 12 months	76.3 ± 19.7	76.6 ± 19.0	0.936
KSS 12 months	94.6 ± 5.7	91.7 ± 9.5	0.091

**Table 9:** Subgroup analysis of changed CPAK with minor group change (adjacent group changes)

	Changed CPAK (n = 18)	Same CPAK (n = 50)	*p* value
Mean ± SD			
KSFS 6 weeks	56.3 ± 23.1	44.2 ± 25.9	0.166
KSS 6 weeks	87.2 ± 8.8	85.5 ± 10.5	0.606
KSFS 3 months	74.0 ± 17.6	59.5 ± 23.9	0.087
KSS 3 months	91.6 ± 5.1	88.6 ± 12.1	0.450
KSFS 6 months	70.8 ± 24.8	68.2 ± 19.3	0.712
KSS 6 months	93.5 ± 5.5	90.0 ± 12.0	0.339
KSFS 12 months	75.4 ± 28.0	76.6 ± 19.0	0.880
KSS 12 months	95.3 ± 3.4	91.7 ± 9.5	0.067

**Table 10:** Subgroup analysis of changed CPAK with major group change (non-adjacent group changes)

	Changed CPAK (n = 134)	Same CPAK (n = 50)	*p* value
Mean ± SD			
KSFS 6 weeks	52.0 ± 24.8	44.2 ± 25.9	0.130
KSS 6 weeks	85.6 ± 13.4	85.5 ± 10.5	0.961
KSFS 3 months	63.0 ± 24.0	59.5 ± 23.9	0.500
KSS 3 months	87.9 ± 10.9	88.6 ± 12.1	0.763
KSFS 6 months	72.2 ± 19.5	68.2 ± 19.3	0.306
KSS 6 months	93.0 ± 8.1	90.0 ± 12.0	0.177
KSFS 12 months	77.0 ± 19.4	76.6 ± 19.0	0.913
KSS 12 months	94.2 ± 6.6	91.7 ± 9.5	0.162

CPAK changes were then classified by coronal planes as well as minor and major change groups. Minor CPAK change is defined as adjacent group changes while major CPAK changes suggest non-adjacent group changes [16]

## References

[1] Li D, Li S, Chen Q, Xie X. The prevalence of symptomatic knee osteoarthritis in relation to age, sex, area, region, and body mass index in china: a systematic review and meta-analysis. Front Med (Lausanne). 2020;7:304. 10.3389/fmed.2020.00304. 32766258 10.3389/fmed.2020.00304 PMC7378378

[2] Tang X, Wang S, Zhan S, et al. The prevalence of symptomatic knee osteoarthritis in China: results from the China Health and retirement longitudinal study. Arthritis Rheumatol. 2016;68(3):648–53. 10.1002/art.39465. 26474054 10.1002/art.39465

[3] Yelin E, Weinstein S, King T. The burden of musculoskeletal diseases in the United States. Semin Arthritis Rheum. 2016;46(3):259–60. 10.1016/j.semarthrit.2016.07.013. 27519477 10.1016/j.semarthrit.2016.07.013

[4] Cui A, Li H, Wang D, Zhong J, Chen Y, Lu H. Global, regional prevalence, incidence and risk factors of knee osteoarthritis in population-based studies. EClinicalMedicine. 2020;29–30:100587. 10.1016/j.eclinm.2020.100587. 34505846 10.1016/j.eclinm.2020.100587 PMC7704420

[5] Sharma L, Song J, Felson DT, Cahue S, Shamiyeh E, Dunlop DD. The role of knee alignment in disease progression and functional decline in knee osteoarthritis. JAMA. 2001;286(2):188–95. 10.1001/jama.286.2.188. 11448282 10.1001/jama.286.2.188

[6] Siddiqi A, Horan T, Molloy RM, Bloomfield MR, Patel PD, Piuzzi NS. A clinical review of robotic navigation in total knee arthroplasty: historical systems to modern design. EFORT Open Rev. 2021;6(4):252–69. 10.1302/2058-5241.6.200071. 34040803 10.1302/2058-5241.6.200071 PMC8142596

[7] MacDessi SJ, Griffiths-Jones W, Harris IA, Bellemans J, Chen DB. Coronal Plane Alignment of the Knee (CPAK) classification. Bone Joint J. 2021;103B(2):329–37. 10.1302/0301-620X.103B2.BJJ-2020-1050.R1. 10.1302/0301-620X.103B2.BJJ-2020-1050.R1 PMC7954147 33517740

[8] Agarwal S, Ayeni FE, Sorial R. Impact of change in coronal plane alignment of knee (CPAK) classification on outcomes of robotic-assisted TKA. Arthroplasty. 2024;6:15. 10.1186/s42836-024-00239-1. 38570879 10.1186/s42836-024-00239-1 PMC10993496

[9] Al-Abbasi G, Wallace D, Mahmood F, Ohly N, Clarke J. Does change in coronal plane alignment of the knee classification following total knee arthroplasty influence patient-reported outcomes and survivorship? A review of 1,062 cases with ten years’ follow-up. Orthop Procs. 2024;106B(SUPP17):2–2. 10.1302/1358-992X.2024.17.002.

[10] Konishi T, Hamai S, Tsushima H, et al. Pre- and postoperative coronal plane alignment of the knee classification and its impact on clinical outcomes in total knee arthroplasty. Bone Joint J. 2024;106B(10):1059–66. 10.1302/0301-620X.106B10.BJJ-2023-1425.R1. 10.1302/0301-620X.106B10.BJJ-2023-1425.R1 39348894

[11] Dall’Oca C, Ricci M, Vecchini E, et al. Evolution of TKA design. Acta Biomed. 2017;88(2S):17–31. 10.23750/abm.v88i2-S.6508 10.23750/abm.v88i2-S.6508 PMC6178992 28657559

[12] Okazaki K. Adopting the joint line theory for bone resection in cruciate-retaining total knee arthroplasty to prevent flexion gap tightness. Orthop Surg. 2022;14(5):984–9. 10.1111/os.13256. 35434965 10.1111/os.13256 PMC9087447

[13] Dhungana H, Jangid S, Goyal M. Alignment techniques in total knee arthroplasty: Where do we stand today? Chin Med Sci J. 2024;39(3):217–25. 10.24920/004372. 39099407 10.24920/004372

[14] Blakeney W, Clément J, Desmeules F, Hagemeister N, Rivière C, Vendittoli PA. Kinematic alignment in total knee arthroplasty better reproduces normal gait than mechanical alignment. Knee Surg Sports Traumatol Arthrosc. 2019;27(5):1410–7. 10.1007/s00167-018-5174-1. 30276435 10.1007/s00167-018-5174-1

[15] Wang SP, Wu PK, Lee CH, Shih CM, Chiu YC, Hsu CE. Association of osteoporosis and varus inclination of the tibial plateau in postmenopausal women with advanced osteoarthritis of the knee. BMC Musculoskelet Disord. 2021;22(1):223. 10.1186/s12891-021-04090-2. 33632177 10.1186/s12891-021-04090-2 PMC7908654

[16] Kim SE, Yun KR, Lee JM, et al. Preserving coronal knee alignment of the knee (CPAK) in unicompartmental knee arthroplasty correlates with superior patient-reported outcomes. Knee Surg Relat Res. 2024;36:1. 10.1186/s43019-023-00204-3. 38167246 10.1186/s43019-023-00204-3 PMC10763258

[17] Writing Committee, Pangaud C, Siboni R, et al. Restoring the preoperative phenotype according to the coronal plane alignment of the knee classification after total knee arthroplasty leads to better functional results. J Arthroplast. 2024;39(12):2970–2976. 10.1016/j.arth.2024.06.012 10.1016/j.arth.2024.06.012 38880407

[18] Luo CF. Reference axes for reconstruction of the knee. Knee. 2004;11(4):251–7. 10.1016/j.knee.2004.03.003. 15261208 10.1016/j.knee.2004.03.003

[19] Corban LE, van de Graaf VA, Chen DB, Wood JA, Diwan AD, MacDessi SJ. How often do we alter constitutional limb alignment, joint line obliquity, and Coronal Plane Alignment of the Knee (CPAK) phenotype when performing mechanically aligned TKA? Bone Jt Open. 2024;5(2):109–16. 10.1302/2633-1462.52.BJO-2023-0122. 38325412 10.1302/2633-1462.52.BJO-2023-0122 PMC10849801

[20] Sappey-Marinier E, Batailler C, Swan J, et al. Mechanical alignment for primary TKA may change both knee phenotype and joint line obliquity without influencing clinical outcomes: a study comparing restored and unrestored joint line obliquity. Knee Surg Sports Traumatol Arthrosc. 2022;30(8):2806–14. 10.1007/s00167-021-06674-w. 34291311 10.1007/s00167-021-06674-w

[21] Insall JN, Binazzi R, Soudry M, Mestriner LA. Total knee arthroplasty. Clin Orthop Relat Res. 1985;192:13–22. 3967412

[22] Patil S, McCauley JC, Pulido P, Colwell CW Jr. How do knee implants perform past the second decade? Nineteen- to 25-year followup of the Press-fit Condylar design TKA. Clin Orthop Relat Res. 2015;473(1):135–40. 10.1007/s11999-014-3792-6. 25082622 10.1007/s11999-014-3792-6 PMC4390935

[23] Abdel MP, Parratte S, Blanc G, et al. No benefit of patient-specific instrumentation in TKA on functional and gait outcomes: a randomized clinical trial. Clin Orthop Relat Res. 2014;472(8):2468–76. 10.1007/s11999-014-3544-7. 24604110 10.1007/s11999-014-3544-7 PMC4079860

[24] Hood B, Blum L, Holcombe SA, et al. Variation in optimal sagittal alignment of the femoral component in total knee arthroplasty. Orthopedics. 2017;40(2):102–6. 10.3928/01477447-20161108-04. 27841930 10.3928/01477447-20161108-04

[25] Kazarian GS, Haddad FS, Donaldson MJ, Wignadasan W, Nunley RM, Barrack RL. Implant malalignment may be a risk factor for poor patient-reported outcomes measures (PROMs) following total knee arthroplasty (TKA). J Arthroplast. 2022;37(6S):S129–33. 10.1016/j.arth.2022.02.087. 10.1016/j.arth.2022.02.087 35248754

[26] Migliorini F, Maffulli N, Cuozzo F, et al. Mobile bearing versus fixed bearing for unicompartmental arthroplasty in monocompartmental osteoarthritis of the knee: a meta-analysis. J Clin Med. 2022;11(10):2837. 10.3390/jcm11102837. 35628963 10.3390/jcm11102837 PMC9143434

[27] Benazzo F, Jannelli E, Ivone A, et al. Knee arthroplasty system with medialized keel: seven-year follow-up of a pioneer cohort. Knee. 2020;27(3):624–32. 10.1016/j.knee.2020.04.014. 32563416 10.1016/j.knee.2020.04.014

[28] Migliorini F, Pilone M, Schäfer L, Simeone F, Bell A, Maffulli N. Functional alignment in robotic-assisted total knee arthroplasty: a systematic review. Arch Orthop Trauma Surg. 2024;144(4):1741–9. 10.1007/s00402-023-05195-0. 38337093 10.1007/s00402-023-05195-0

[29] Shatrov J, Battelier C, Sappey-Marinier E, Gunst S, Servien E, Lustig S. Functional alignment philosophy in total knee arthroplasty - rationale and technique for the varus morphotype using a CT based robotic platform and individualized planning [published correction appears in SICOT J. 2022;8:18. 10.1051/sicotj/2022017]. SICOT J. 2022;8:11. 10.1051/sicotj/2022010 10.1051/sicotj/2022010 PMC8973302 35363136

[30] Parsons T, Al-Jabri T, Clement ND, Maffulli N, Kader DF. Patella resurfacing during total knee arthroplasty is cost-effective and has lower re-operation rates compared to non-resurfacing. J Orthop Surg Res. 2021;16(1):185. 10.1186/s13018-021-02295-8. 33706779 10.1186/s13018-021-02295-8 PMC7948323

